# 92 Design of a Nursing Morbidity and Mortality Conference

**DOI:** 10.1093/jbcr/iraf019.092

**Published:** 2025-04-01

**Authors:** Emily Werthman, Christine Steen

**Affiliations:** Johns Hopkins Burn Center; Johns Hopkins Bayview Medical Center

## Abstract

**Introduction:**

The process of presenting a case for morbidity and mortality cases is often the first step of the loop closure process, the cornerstone of a quality and performance improvement program. At present, most burn centers do not include, or minimally include, nurses as part of the morbidity and mortality process, even when nursing errors or processes are part of the case presentation. It was our goal to design a new system for nursing to report cases and identify areas for improvement, called “case conference.” The goal of case conference was to use the identified AFIs to inform educational offerings, loop closure needs, and provide a forum for nurses to present cases in a multi-disciplinary forum.

**Methods:**

Per the burn center’s performance improvement plan, all mortalities are presented by burn fellows to surgical M&M. As a result, case conference sought to focus on complications that might not warrant escalation to surgical M&M presentation, i.e. deviation from nursing protocol that resulted in a stage II hospital acquired pressure injury. Morbidities considered for case conference presentation include complications the burn dictionary including, but not limited to: non-compliance with burn activation, self-extubation, hypothermia, failure to implement protocol for hypoglycemia, documentation issues (nursing), failure to establish enteral nutrition within 4 hours of admission, deep vein thrombosis (DVT), hospital acquired pressure injury, unplanned intubation, catheter associated urinary tract infection (CAUTI), central line associated blood stream infection (CLABSI), and ventilator associated pneumonia (VAP). Whenever possible, the primary nurse on the day of the complication is tasked with presenting the case at case conference. Initially, case conferences were held on an as needed basis. Morbidities to be presented are determined by the burn program coordinator, in collaboration with the medical director and patient care manager.

**Results:**

As a new practice, we cannot compare to previous cases wherein only surgical M&M was used to discuss complications. However, case conferences have yielded crucial conversations about nurse driven fluid resuscitation and pain management protocols.

**Conclusions:**

Although not yet common place throughout burn centers, we encourage the inclusion of nursing in the morbidity review process either through independent review, like our case conference, or through integration in existing M&M processes.

**Applicability of Research to Practice:**

Involving nursing in presentation of morbidities in a multi-disciplinary format provides important data related to performance improvement and loop closure. It also provides nurses with a professional development opportunity to hone their presentation skills and provide relevant data points. Combing information obtained from both case conference and M&M provides a fuller understanding of cases, allowing for better loop closure.

**Funding for the Study:**

N/A

